# Æsthetic Application of Dental Art

**Published:** 1886-02

**Authors:** W. Austin Currie

**Affiliations:** Instructor in Carving and Plastic Manipulation, Boston Dental College


### SELECTIONS.

### The Æsthetic Application of Dental Art.

BY W. AUSTIN CURRIE, D.D.S., INSTRUCTOR IN CARVING AND PLASTIC MANIPULATION, BOSTON DENTAL COLLEGE.

Read before the Annual Association of the Boston Dental College, Boston, October 13, 1885.

On different occasions during my period of practice, I have been requested by patients to explain why it is that dentists seem to know so little about art. What can I say, more than to confess that I do not know? I have found repeatedly that such a reply does not in any sense afford satisfaction to the inquirer, or tend to extricate me from an embarrassing position, but only serves to push me more deeply into difficulties. The query in the outset was prompted, probably, by a passing thought, and had they received any convincing reply, their momentary inquisitiveness would have been gratified and the subject dropped then and there. But, perceiving my inability to give any reason why a profession so entirely made up of artistic requirements, should have no facilities for cultivating an æsthetic taste in the dental colleges, their curiosity naturally became aroused, and I regretted that I allowed them to see that the question was too much for me.

And can we wonder greatly that such questions meet us, when we reflect that one of the two more important branches of dentistry consists entirely in restoring nature by art? What is art? Of all the short words in our language there are but few which are handled more frequently, and that, too, by persons who really have but a faint idea of what those three letters signify. How many of the thousands of sightseers who visit the galleries of our own and foreign countries, and are thus permitted to look upon the work of celebrated artists, comprehend that the creations before them are the result of the careful study of a lifetime. They are only conscious of the general effect of a work of art, and do not realize what a vast amount of study and labor is required to attain such grand results.

For the sake of illustration, let us for an instant glance at a painting. The artist has presented his work true to life; almost too much so to be pleasant. It has been truly said, that, next to the actual pain experienced in being a party to sorrow is the touching suggestion which a faithful picture communicates. Wandering over the canvas, our eye first notes the general interest; we remark the excellency of the drawing, the perspective, and the coloring, but soon our attention becomes riveted on the central object, a representation of an old woman, a glance at whose face at once proclaims the *special* motive of the work. The scene is a court of justice, with all of its characteristic stiffness and its unpleasant suggestiveness. Spectators are there with faces full of feeling. The learned lawyers, in powdered wigs, are there true to life, while, in contrast, stands this poorly-dressed woman before the judge who is about to pass sentence of death upon an only son. She is offering a mute appeal—no words of supplication; but the trembling arms and old withered hands outstretched, tears flowing over wrinkled cheeks, the intense agony so plainly depicted on every feature, is an appeal more touching than any eloquence the tongue could have offered. Such a picture commands the attention of every beholder; but how few realize what an immense amount of work it required to reach such a mark of perfection. What an insight into human nature was called for to enable the artist to portray this touching scene. Do they reflect upon the fact that a knowledge of anatomy, physiology, and other studies are required, as well as of colors and their application? Many persons have a faculty of painting a fair representation of the human face upon canvas—they are said to be talented in that respect. But if they expect to approach anywhere in the vicinity of perfection, they must be acquainted with the forms of the bones of the head, as well as the muscles and softer tissues. How can they truthfully give the form of the outside without some knowledge of the framework within.

Now, if this knowledge is indispensable to an artist, why should it not be equally desirable that the mechanical dentist should be conservant with its principles also? An artist, by making himself intimately acquainted with the human form, and by his perceptiveness; power of imitation, and deftness of hand, can place a representation of it upon canvas. He can counterfeit any feature and bring forward any expression. If his work be sculpture, he does away with deception by placing actual shape before us. The one deceives the eye by light and shade, the other give us substance, the natural light contributing the appearance by which we distinguish the different forms of objects. With the dentist, the object is the same; but, unlike the former, he has not blank canvas or hard stone to work upon, but has living tissues on which he can learn to put different expressions. Many will say that expression comes from the mind, and that we have no control over it except in our own features. True, it comes from the mind, but it shows itself upon the face, and different expressions are but the result of the action of different muscles in moving portions of the skin (and underlying tissues) so that a new light strikes upon it; this varying light, with its consequent shade, adds a new aspect to the countenance.

An artist can change the whole expression of a painted face by the addition of a little extra light and shade, and that with but a few strokes of the brush. All he does is to *apparently* raise some portions with light and cause others to recede by rendering them a little darker. If colors will change the countenance by appearing to raise portions, is it not patent that *actually* raising those parts will do it even better. It takes but the slightest change in position of the lips and adjacent parts to make a great change in expression. You will put greater faith in this assertion if you will experiment a little yourselves. Take a portrait, or, better, two, precisely alike. The lithographs, such as are exhibited in the street-windows by theatres will answer every purpose. It will be best if we choose a lady's face, as it is with the ladies' features, principally, that we will wish to utilize our knowledge, if it be found worthy of use. It is, we will say, a full face, and one with a pleasant look about the features. Now with a crayon make a short downward mark from each corner of the mouth on one picture only, as we will need the other for comparison; then with your finger-end rub in a little white just under the lower lip, adding a trace of shade to the upper lip in the same manner. What is the result? You have a face the owner of which you would think had not an earthly friend. Now, after erasing what you have added, reverse the order of things by placing the light on the upper, the shade on the lower, and drawing your lines upward from angle of mouth, instead of down, as before, and you will find the result reversed also: for the face is found now to be bright and cheerful. By going a little further, and placing two curved lines from base of nose downward, past corners of mouth, with convexity outward, you have a grin that will often prove contagious. A thin piece of paper placed under the lip on the alveolar ridge, will add its quota to the expression.

These changes have been effected by a few marks that would be hardly noticeable anywhere else on the drawing, unless about the eyes. If this can be done on paper, can it not be accomplished, say in part, at least, with the face itself? Of course, it is an entirely different matter when we come to put our theory into practice, as it will require that we educate the eye by long and close observation of nature, and teach the hands to follow its dictates. The lips, of all the features, are the most direct index to the feelings, and the expression characteristic of every face is due almost entirely to the effect of light and shade about the lips. It is (I believe) because we forget this fact, that we often feel dissatisfied with our work even when the patient expresses pleasure with it. I believe, too, that it is owing to the fact that the profession shows want of knowledge, care, or ability in this respect that we meet with so many faces whose owners wear features that are blank, or almost expressionless about the mouth. When we hear such common expressions as a beautiful smile, or eloquent lips, do we ever ask ourselves in what does this beauty or that eloquence consist?

Our course of studies in mechanics is, to the best of my knowledge, perfect as far as it goes; but is it carried out to its greatest extent? For instance, we are told to carefully note the play of the lips over the wax articulation; but *what is it* in the play of the lips that we are to study? I can but answer for myself. I was expecting to hear *expression* treated in every detail, and felt disappointed when, upon nearing a situation where I felt genuine interest, I found that the points which seemed to me of the utmost importance were passed over with a casual remark or two. I know when I say this that no one will infer that I am trying to pit my limited knowledge and experience against years of practical work of my instructors. Perhaps our teachers expected the students to understand without further instruction. Perhaps my classmates, by superior ability, were enabled to bridge over what seemed to me a break in the most interesting part of our course, or it may that I am expecting too much of dentistry.

Now, the question is, how to proceed in order to derive any professional benefit from the ideas here suggested. As I regard it, the first requisite is to study nature, and study closely. As far as the human countenance is concerned, no one has more opportunity for observation than the dentist. Our business carries with it a certain license, so that we can examine a patient's features critically without giving offence. Unlimited chances are thus offered, and we can watch every face that we work over, and then we must analyze what we see.

Note the general expression, the fulness of the lips, and the tooth development beneath them. Watch where and how the light strikes the face, and what is the result of different lights upon the same features. If any teeth are absent, note the effect upon the expression. Observe color of hair, eyes, and complexion, and then examine the exact shade of the teeth, also the size and shape of the teeth as compared with size and shape of features, form of head, and general build of body. This can be done by a trained eye, while the patient undergoes the usual examination without suspecting but that your undivided attention is given to the teeth. Then compare this with the next face that presents itself. If this is to afford any benefit to the practitioner, the observation and comparison must be critical. We are too apt to bestow a casual glance, and forget as soon as the patient is gone, whereas we should memorize or make a note of the most important points.

To make ourselves *masters* of the art of light and shade, we must go even further than this. *We must cultivate a taste for art.* We subscribe for some one or perhaps all of the dental journals—why not for an art journal as well? We try to keep ourselves posted on all the new ideas in our profession—why not mix up a little art with them? Suppose that we cannot see at first that it is benefiting our work, we cannot deny that it is refining, as well as affording a pleasant change from an occupation that is in many respects disagreeable. Then when we have leisure we can turn our hands to a little modelling.

If we have a small space in our laboratories, why not utilize it by making a bench in a corner that has a good light, and then investing in a few pounds of artists' clay and one or two modelling tools. By an hour's work and the outlay of a few dollars, and we have a way of passing our spare moments that will be novel and instructive. For it is in modelling and casting that the effect of light and shade is made most manifest. If we have doubts of our ability to reap any benefit from modelling, why not try casting, choosing a friend or a patient's face for a subject? Suppose that we try it in this way, our subject being a person who wears a full denture and with a bare face? First make a mould of the face (wax will answer) with the mouth empty. Then one with the plates in position, and then, after building up with wax in different places on the plates, place them in the mouth and make a third mould. Now to cast with plaster. The wax mould will of necessity be thin, so that it is best before casting to build up around the outside of the mould with sand so that the weight of the plaster, when soft, will not change the shape. Then if you will tint the water with powdered or dry umber and a little vermilion before you mix the plaster, the latter will more nearly resemble the flesh-color. After casting all three, and allowing the plaster to set, if you will remove the wax from each you will be surprised at the entirely different expression on each face. The question is, where you will find a person who will submit to so much trifling? I have experimented with many different faces, and thus far they have been so deeply interested in the novelty of the work that I have yet to meet the first complaint. In conclusion, let me say that I believe that if dental practitioners who have never tried this, would do so, they would be surprised and pleased with its effect upon their laboratory work. At first, I became discouraged, and thought that I failed more often than I succeeded; but now, when I look back I find that I have not failed. On the contrary, I have succeeded so far that now I am where I can see what can be accomplished if one is perseverant. I am convinced that the study of facial expression has helped me wonderfully, and has given a new impetus to work that before seemed monotonous. One thing in which I think all will agree with me is this, that a little art cannot injure us in any way, even if it is æsthetic.—*Journal Am. Med. Asso.*

